# An assessment of Nigeria's health systems response to COVID-19

**DOI:** 10.4314/gmj.v56i3s.9

**Published:** 2022-09

**Authors:** Chinyere Okeke, Benjamin Uzochukwu, Chioma Onyedinma, Obinna Onwujekwe

**Affiliations:** 1 Health Policy Research Group, College of Medicine, University of Nigeria, Enugu Campus, Enugu, Nigeria; 2 Department of Community Medicine, University of Nigeria Enugu Campus, Enugu, Nigeria; 3 Department of Health Administration and Management, University of Nigeria Enugu Campus, Enugu, Nigeria

**Keywords:** COVID-19, Health systems response, strengthening health systems, Service delivery, Nigeria

## Abstract

**Objectives:**

This study aims to understand and report on selected health system interventions considered nationally and sub-nationally of particular significance both in terms of COVID-19 responses and in strengthening the health system for the future.

**Design:**

A review of published and grey literature, including journals, news/ media and official documents, was conducted from 1st December 2019 to 31st December 2020. The reviewers read and extracted relevant data using FACTIVA in a uniform data extraction template. Responses that related to service delivery were captured.

**Setting:**

The assessment considered responses at the national and two state levels: Lagos and Enugu, representing the epicentre and a low COVID-19 burden centre.

**Inclusion criteria:**

Documents and news that mentioned COVID-19 response, particularly service delivery aspects, were included in this review.

**Results:**

The identified interventions were mostly technical support targeted at health workers: including training of about 17,000 health workers, supervising and engaging more health workers, upgrading laboratories and building new ones to improve screening and diagnosis, and motivation of health workforce with incentives. Furthermore, the influx of philanthropic contributions improved the data and information systems supply of medicines, medical products and non-pharmaceutical protective materials through local production. The presence of political will and the government's efforts in health system's response to COVID-19 facilitated these interventions.

**Conclusions:**

Interventions of state and non-state actors have strengthened the health systems to some extent. However, more needs to be done to sustain these gains and make the health system resilient to absorb unprecedented shocks.

**Funding:**

IDRC Canada Grant # 109479-001

## Introduction

Nigeria's Health system comprises all people, institutions, resources and activities whose primary purpose is to promote, restore and/or maintain health, while health systems strengthening are strategies, interventions and activities designed to sustain health system performance.^1^ A strong health system is characterised by its ability to respond to emergencies while remaining resilient in promptly delivering high-quality routine essential services.^2^ This is not the case in most low- and middle-income countries, such as Nigeria, thus making them very vulnerable to the COVID-19 pandemic. Before the pandemic, in most cities, health infrastructures were dilapidated as they had not received adequate attention.^3^

Nigeria's weak health system and poor health indices made the country very vulnerable to this outbreak. According to the WHO, Nigeria was classified as one of the 13 high-risk African countries concerning the outbreak.^4^ Looking at the health situation in Nigeria, a report from WHO pronounced that almost ten per cent of newborn deaths globally in 2016, occurred in Nigeria.^5^ Furthermore, five countries accounted for half of all newborn deaths, and Nigeria was third on that list.^6^ According to UNICEF, Nigeria is responsible for a high number of under-five child deaths globally.^7^ An average of 20,000 Nigerians travel to India each year for medical treatment due to the absence of a robust healthcare system at home.^7^ Nigeria's public health system is riddled with poor coordination, lack of accountability, little or no incentives to improve performance and lack of resources at the frontline.^8^ Whilst Nigeria was dealing with all these, the fight against the COVID-19 pandemic began.

According to WHO, health systems are responsible for delivering services that improve, maintain, or restore the health of individuals and their communities. These include the care provided by hospitals and family doctors, prevention and control of communicable diseases, health promotion and health workforce planning.^9^ With the advent of the COVID-19 pandemic, regional, national, and local efforts were made by government and non-governmental agencies to respond to the pandemic. Some of this health systems preparedness followed lessons from previous epidemics like the Ebola Virus Disease epidemic.^10^,^11^ Most of these activities impacted the health systems, with some leading to strengthening the country's health systems. Without proper documentation of measures taken at the national level and noting which aspects of the health systems were strengthened, it will be difficult to recognise and replicate such efforts.

This paper examines the extent to which the health response to COVID-19 in Nigeria has strengthened the health systems, and identifies the significant interventions that contributed nationally and sub-nationally in terms of COVID-19 responses as well as strengthening the health system for the future. The findings of this review would help policymakers, investors, development partners, civil society organisations, non-governmental organisations and academics in Nigeria to reflect on ways to adopt, adapt and scale up the identified positive measures when the need arises or when resources are available. Historically pandemics are recurrent, and there is thus the need to review the available evidence, capacities and measures to prepare for the future. It is also important to identify the lessons to be learned from this first phase of the pandemic that can inform health system responses during this ongoing COVID-19 and for future epidemic prevention and management.

## Methods

### Study design

This study was undertaken using a rapid assessment of national and subnational published and grey literature, including journals, news/ media documents and official documents published from 1st December 2019 to 31st December 2020.

### Data sources: Document search and retrieval

The search for electronic documents and grey literature was performed from October 2020 to January 2021. Published government documents were retrieved from relevant organizational websites such as NCDC, PTF, Federal Airports Authority of Nigeria (FAAN). The government documents were guidelines/ protocols, reports and plans written in English. We used FACTIVA to retrieve online media reports from major newspapers (Vanguard, Punch, Guardian, The Nation, Business Day, and Premium Times) that reported national and State level health system responses to COVID-19. We also assessed other online news sources consistently reporting health systems response to COVID-19 in Nigeria. Some of these were Nigeria Health Watch, Apo Africa Newsroom, Federal ministry of health (FMoH), media, TalkNaija, International Development News (Devex) and PricewaterhouseCoopers (PwC) Nigeria.

Research articles were sourced from online journals written in English and published from December 2019 to December 2020. The geographical scope was the national and sub-national levels in Nigeria. The search terms/queries included: “COVID-19”, “Nigeria”, “Lagos”, “Enugu”, “policies”, “service delivery”, “health”, “infrastructure”, “medicines and technologies”, “health financing”, “health workforce”, “data management”, “research” etc. These were generated using combinations of primary keywords or secondary keywords. The search was conducted in English and performed in PubMed, Google Scholar and Scopus. The decision to use Lagos and Enugu represented a high burden (Epicentre) and a low-burden area in the country.

### Data collection/extraction

The rapid assessment was performed by two persons who sourced documents independently and merged it following removing all duplicated documents. The reviewers read and extracted relevant data using a uniform data extraction template. The template was structured in themes using the health system building blocks and subthemes that captured the state and non-state actors. The template captured the following headings: i) source of document including web address where relevant; ii) full citation of the document; iii) scope of the document – national or subnational; iv) Building block(s) – leadership and governance, health care financing, service delivery, Health management information system, Human resource for health, Medicines and technology, Partnership for health; v) findings on health systems interventions during COVID-19. A repository of all documents, journals and media reports was generated.

### Data analysis

A standard operating procedure was developed during data extraction and analysis. The data extracted by each assessor were synthesised across documents and media reports for each thematic area to generate a comprehensive transcript of document review findings for each thematic area. The thematic transcript from the document assessment was summarised through narrative synthesis performed along the main themes that emerged, and the summary reports were merged. Analysis was conducted through the manual thematic content method.

Responses targeted to help improve health systems were further analysed in themes of technical support and interventions targeted at health workers. The sub-themes that emerged were: training and supervision, screening and diagnosis, human resource management and incentives, infrastructure, equipment and resources, improved data and information systems, and supply of medicines, medical products and non-pharmaceutical preventive materials. Content summary and re-categorization were done where misclassification was observed from the initial categorization.

### Data Presentation

The identified interventions and strategies that have affected the health systems were identified and extracted using the following headings: article name, journal/lead organization/author, publication details, source/URL, type of document and coverage (national or state), the main purpose of the document, does it specify a health response to COVID-19? If so, what? Which of the health system building blocks was referred to? how is it likely to strengthen health system for future epidemics?

### Ethical Clearance

The University of Nigeria Teaching Hospital Ethics Committee approved the study protocol. NHREC/05/01/2008B-FWA00002458-1RB00002323.

### Analytical Framework for this study

The WHO health systems building block was developed in 2007. This approach highlights the dynamic architecture and interconnectedness of the health system building blocks and the role of people at the centre of the health system as mediators, beneficiaries, and drivers of the system.^12^

The various blocks are a) Governance: which deals with strategic policy frameworks combined with effective oversight, coalition building and regulation. b) Service delivery: to provide effective, safe, quality personal and non-personal health interventions to those that need them. c) Health workforce: to be responsive, fair and efficient to achieve the best health outcomes possible, given available resources and circumstances. d) Health information system: production, analysis, dissemination and use of reliable and timely information on health data. e) Medicines and technologies: provide equitable access to essential medical products, vaccines and other technologies of assured quality, safety, efficacy and cost-effectiveness.

f) Health care financing: adequate funds for health in ways that ensure people can use needed services and are protected from financial catastrophe.^12^

This study identified all the COVID-19 responses based on the building blocks. Though all building blocks are interconnected eg service delivery with building the capacity of the health workforce, using quality data and medical supplies. Thus, the analysis was based on this, and all interventions and responses in these areas were captured.

## Results

The identified health systems responses from the documents in Table 1 were through technical support and interventions targeted at health workers. These included training and supervision, screening and diagnosis, human resource management and incentives (financial and non-financial), equipment and resources, improved data and information systems, and supply of medicines, medical products and non-pharmaceutical preventive materials through local production and partnerships.

**Table 1:** Summary of the type of documents assessed and the interventions identified

Type of Document	Number of items	Health system responses/interventions identified
Government documents	9	From all 9 documents
Media documents	171	From all 171 documents
Journal articles	18	From all 18 documents
**Total**	**198**	

Overall, the role of political will and the government's efforts in health systems response to COVID-19 facilitated these interventions.

### Technical support, training and supervision

Training of health workers appeared to be a recurrent theme among the documents reviewed. Personnel with experience in emergency response in epidemics, such as polio emergency operations centre members, were engaged at the onset of the pandemic and trained by the Federal Ministry of Health (FMoH) on emergency preparedness and disease outbreak response.^13^

There was also the production of case management protocol and guidelines for the country's response, which was revised severally as the pandemic evolved, and new knowledge about this novel disease emerged. The FMoH then worked with State governments in training health personnel at the State level. They included sample collectors at General hospitals and primary health centres (PHC) for achieving at least one sample collection site at every local government area (LGA), with efficient sample retrieval logistics to convey samples to laboratories; 176 intensive care specialists and biomedical engineers were trained on how to use or maintain the oxygen concentrators and ventilators purchased/received from donors and distributed to the hospitals across the federation.^14^,^15^

The Nigeria Centre for Disease Control (NCDC) trained and mentored State Emergency Operations Centre (EOC) team health workers on the incident management system as a tool for outbreak response coordination, development and operationalisation of data management, analysis and use.^16^ They also activated a national public health emergency operations centre (PHEOC) in response to the COVID-19 pandemic in March 2020 in the Federal Capital Territory (FCT)-Abuja and Lagos State where the index case of COVID-19 was detected.^17^ At that time, only a handful of states were able to activate a COVID-19 PHEOC due to a lack of resources such as steady internet access, capacity, and knowledge, but now, COVID-19 PHEOCs have been activated across all 36 Nigerian states plus the Federal Capital Territory (FCT)-Abuja. They also trained primary healthcare workers on infection prevention and control and made these available online for easy retraining of health workers at their pace. The emergence of a strengthened Nigeria Centre for Disease Control (NCDC) at national and sub-national levels has enhanced the country's diagnostic and surveillance capacity for infectious diseases.18 The establishment of community practice for COVID-19 case managers and deployment of online IPC training program for healthcare workers across the country helped strengthen the country's preparedness towards service delivery in response to COVID-19.^16^

### Screening/Diagnosis

At the onset, Nigeria adopted the Screen, Isolate and Notify (S-I-N) COVID approach for early recognition and source control for IPC in health facilities or hospitals.^17^ Initial measures instituted by the National Coronavirus Preparedness Group (NCPG) included strengthening in-country diagnostic capacity to test COVID-19 by leveraging and optimising three existing laboratories within the NCDC molecular laboratory network. In June 2020, Nigeria's capacity for COVID-19 testing was about 2,500 samples daily.^19^ However, only 50 per cent of this capacity was utilised due to constraints in the availability of testing kits, laboratories, and human resources. As at 5th of July, 2021, the total number tested for the virus was 2,331,734, representing only 0.012 per cent of the Nigerian population in a country of about 200 million. But currently, 163 Polymerase chain reaction laboratories across 35 states of the federation and the federal capital territory (FCT) exist as shown in Table 2.^20^

**Table 2:** Summary of Screening/diagnosis improvement in the country

Screening/diagnosis activity	June 2020	July 2021
Number of people tested for COVID-19 out of 210,000,000	115,760 persons	2,331,734 persons
Capacity for COVID-19 tests per day	2,500 samples per day	5,250 samples per day
Number of PCR laboratories and testing centres in country	12 government only	85 government, 69 private and 9 corporate labs

To increase the testing capacity of states, the NCDC co-opted private laboratories to achieve this target. This was also adopted at the state levels, increasing testing by over 40,000 in one month.^21^ However, these private laboratories had to meet the criteria listed in the NCDC document to be qualified for certification and to test for COVID-19.^22^

The initial procurement of 107,000 cartridges enabled the use of the available Gene Xpert machines in the country. Then there was the donation of other testing materials like Smart Walkthrough booths for COVID-19 Sample collection by other countries like the Republic of Korea. The NCDC also published new guidance on approved antigen-based RDT for COVID-19 testing in public and private institutions in Nigeria.^23^ This came after the national validation of the 2 antigen RDTs authorized for emergency use by WHO. There was also the deployment of an electronic surveillance system to laboratories to speed up the release of results.

### Human resource management

The health workforce was greatly affected by the pandemic. Through NCDC, the country initially trained and deployed rapid response teams in all 36 states of the federation prior to the detection of the index case. With the detection of the index case on 27th February 2020, trained rapid response teams were deployed to different states, with the state teams leading contact tracing and other response activities.^24^ The polio program staff were also co-opted to assist with the COVID-19 response co-ordination, contact tracing, investigation of cases, risk communication, community engagement and disease surveillance. The support was seen to be very vital as Nigeria was entering the community transmission phase at that time. The NCDC aggressively improved staff capacity in advanced diagnostics, disease surveillance and response. They supported states in training public health workers, set up systems for efficient epidemiological data collection and sharing and established emergency operations.

Cumulatively, over 17,000 HCW were trained across different areas such as infection prevention and control, laboratory operations, and case management by the end of July 2020.

Health workers were motivated by paying COVID allowance for months, and the frontline workers received life insurance coverage.25 Similarly, the Lagos State government went further to motivate health workers managing COVID-19 patients by honouring them with awards and cash gifts for the hard-working ones and also doubled the amount of money stipulated for life insurance for health workers in the state, thus enabling more benefit packages. Deployment of equipment and resources as well as infrastructure

With regards to infrastructure and readiness to bolster-covid-19-response in Nigeria, the existing polio infrastructure was applied.26 However, in a bid to increase testing capacity in the country, the Federal Government built 71 Polymerase chain reaction laboratories across 35 states of the federation and the federal capital territory (FCT). Nigeria had to start building most of the laboratory facilities for sample testing from scratch, as she had less than 15 molecular laboratories across the country, unlike many other countries.^27^

At the preparedness stage and during the pandemic, providing isolation centres was an important strategy adopted globally and specifically equipped for treating infectious diseases. Before the outbreak, Nigeria had only 350 ventilators and 350 Intensive Care Unit (ICU) beds. However, in April 2020, 100 additional ventilators were acquired. An additional 200 ventilators were donated by the government of the United States of America.^28^ Also, ICUs have been provided across various states in the six geopolitical zones of Nigeria. Currently, 112 treatment and isolation centres with oxygen concentrators and ventilators are present in all 36 states and the Federal Capital Territory, with 5,324 beds.^29^ This was also replicated at the subnational level. The provision of such infrastructure will make available facilities for use during future epidemics and enhance service delivery.

Other innovations were the establishment of National Emergency Medical Service & Ambulance System (NEMSAS) by National Council on Health (NCH) to move ill patients to COVID-19 treatment centers.^30^ There was also the purchase of equipment and commencement of free telemedicine response services and phone consultations for patients by most government owned tertiary institutions in the country, to help address their health needs while combating other challenges of the Corona-virus pandemic.^31^

Various items and commodities enhancing service delivery were received as donations from various organizations, locally and internationally. For instance, the Manchester Business School, donated medical equipment to NCDC to aid service delivery in the fight against the COVID-19 pandemic.^32^–^34^ European Union and United Nations handed over medical supplies to Nigeria to aid in their fight against the COVID-19 pandemic.^34^

Similarly, funds were generated quickly for the COVID-19 pandemic response by various government and non-governmental agencies. For instance, the Federal government approved the release of over 49 billion Naira that was part of the 2020 budget, COVID-19 special levy contributions and 12.5 million US dollars in special intervention funds;^35^ 500 billion Naira by Nigeria's Economic Sustainability Plan for COVID-19 Crisis Intervention Fund; over $64 million from the Private Sector Coalition Against COVID-19 Foundation; IMF approved the release of $3.4 billion;^36^ huge donations from partners.

Furthermore, funds were pooled through the launch of the Nigeria/United Nations COVID-19 basket fund that received funds from prominent Nigerians and United Nations agencies and the Oil and Gas Sector Funding Stream coordinated by the Nigerian National Petroleum Corporation (NNPC). Other interventions included 6-month import duty waiver for pharmaceutical companies on medical supplies such as ventilators, test kits, personal protective gear, thermometers, disinfectants, and essential medical consumables.^37^ The pooled funds were released for case detection and risk communication strategies, purchasing PPEs and medical equipment, and building isolation centres. In addition, 126 billion Naira was released for health infrastructure upgrades, purchasing drugs and commodities, laboratory services, Port Health Services and other essential health services.

In March 2020, the Central Bank of Nigeria extended USD 257.9million (NGN100 billion) credit facilities to equip businesses in the health sector to deal with the pandemic's challenges.^38^ These were made available to health product manufacturers, health service providers, pharmaceutical and medical product distributors, and logistics services. It was designed to run from April 2020, till 31st December 2030.^39^

Strengthening health management information systems to generate timely data for health decision-making and service improvement. With the advent of the COVID-19 pandemic, the government channeled so much effort into improving Nigeria's health management information system. This included interventions such as integrating reporting obligations into the existing health information management system by NCDC through the Surveillance Outbreak Response Management and Analysis systems (SORMAS) tool (the trademark of the one being adopted in Nigeria).^40^ There was also the creation of a website and microsite for dissemination of information, as well as the validation of the Nigeria Covid-19 response Action Plan that ensures a single integrated view exists and eliminates unnecessary fragmentations, duplications, and overlaps. It also clarifies adjacencies, and defines responsibilities between the Ministries, Departments and Agencies and the States' Ministries of Health. There was also the launch of a WhatsApp platform by NCDC and an interactive chat box to supply central, verified and current information on COVID-19 and provided a free hotline that is available twenty-four hours daily for calls.^41^ Similarly, other organizations and the private sector also played some part. For instance, the Nigerian National Petroleum Corporation (NNPC) launched an App for COVID- 19 contact tracing while the Private sector set up a program to check health rumors by responding to, dispelling and debunking them.^42^

### Partnerships for health

Nigeria adopted a multi-sectoral response to the COVID-19 pandemic. So many partnerships were formed by various government MDAs, NGOs, and CSOs formed so many partnerships to control the pandemic. The Joint Support Framework takes a whole-of-society and a whole-of-government approach to bring together all sectors and partners operating in Nigeria's COVID-19 response from the national governmental authorities, non-governmental organizations, UN agencies, academic and training institutes, donor agencies, and the affected population.^43^ The current collaborative efforts from the World Health Organization, WHO-AFRO, WAHO, Africa CDC, NCDC, federal and state government health ministries, health institutes, non-governmental organizations and researchers have contributed to the response to service delivery during the pandemic as presently seen. They have helped build the nations' capacities for preparedness and response to the disease, including prompt case identification, diagnosis and use of smart approaches to educate and sensitize the country about the infection. They also provided prompt updated information about the evolving disease and have provided diagnostic materials, with the aim of halting or at least limiting the spread of the infection.

Partnerships also existed with local influencers, such as community and religious leaders, to provide clear messaging on COVID-19 prevention. All these could not have been achieved without Partnership.^42^ The role of political will and government's efforts in health systems response to COVID-19: Political will strongly influence the health sector response to COVID-19. This was expressed in the policies translated into legal mandates that increased public healthcare financing for health systems response to COVID-19. Evidenced by the Institution of a twelve-man Presidential Task Force (PTF) by the president on March 9, 2020 to coordinate a multi-stakeholder response to the pandemic while providing technical and material support to states to manage the outbreak. They worked to oversee and evaluate Nigeria's response strategies and implemented activities daily to curb the virus's spread. Political will was also evidenced by the adjustments made to the national budgets and release of pre-assigned funds as well as the direct support for micro, small, medium enterprises (MSME) involved in health service delivery and the import duty waiver for pharmaceutical companies.^38^

## Discussion

This review has shown that Nigeria has carried out various interventions and strategies targeted at improving service delivery during the COVID-19 pandemic. These included training and supervision of frontline health workers, screening/diagnosis of COVID-19, human resource improvement, deployment of essential equipment and resources, strengthening the health management information system, enhancing partnership and multi-sectoral involvement as well as strong political will as expressed in policies and translated into legal mandates that ensured increased public health care financing for health systems response to COVID-19. However, there are still limitations in the ability of the country to properly deliver quality healthcare services and sustain the gains made during the pandemic.

Experiences from the 2014 Ebola outbreak and Lassa fever helped the country prepare for the COVID-19 outbreak, but only after positive cases were confirmed. There was the Presidential task force at the federal level and the state task force at the State level that helped strategize and implement responses to the pandemic. The NCDC also had an emergency operational centre with an initial plan at only the federal level before expanding to all states.

Despite the influx of philanthropic contributions, which mostly provided palliative measures, most of which will be very difficult to sustain and maintain after the pandemic, there are still gaps concerning strengthening the health systems to respond to emergencies while remaining resilient in delivering high-quality routine essential services promptly. The major gaps identified were lack of isolation centres, PCR labs, well equipped ICUs, as well as challenges to the implementation of contact-tracing and delayed closure of the entry points into the country which could be said to be governance and service delivery issues. The vital response was the development of isolation centres and PCR labs, equipment of ICUs, lockdown to prevent COVID-19 community transmission, initially from 2 states (Lagos the epicentre and Ogun) then to FCT and to all other states.

However, some of these isolation centres and ICUs were makeshift facilities which have been dismantled and did not stay through the lifespan of this pandemic, let alone future epidemics/pandemics. On the other hand, the modern equipped laboratories, ICUs, upgraded health facilities and human resource training will go a long way in helping the country tackle future pandemics, especially as the country is prone to Lassa fever outbreaks, with only three centres that were able to diagnose this prior to COVID-19 but over 71 centres available now. Although the number of pieces of equipment has increased since the advent of COVID-19, they are still not enough to meet the needs of Nigeria's growing population.

Health workers are at the frontline of disease control and prevention but can also be the means and source of nosocomial and community transmission of COVID-19.^44^ Therefore, to mitigate the increasing number of COVID-19 cases requires that health workers adhere to the recommended measures taken to prevent transmission. These measures are affected mainly by knowledge, attitudes, and practices of the frontline workers.^45^ Studies have shown that the training of health workers on infection prevention and control during COVID-19 in Nigeria resulted in excellent knowledge, positive attitude and good practice towards COVID-19.^46^–^48^ However, one of the studies portrayed areas where poor knowledge, negative attitudes and unacceptable practices were observed.^48^ This is similar to findings in other countries^49^ and it highlights the need for continuous public health education of health workers on infectious disease control and prevention, which is why the NCDC has made these training modules available online for health workers' self-refresher courses. At the same time, public health awareness campaigns through risk communication and community engagement activities within the target population of the health workers and host communities need to be sustained to maintain the gains of the training received by the health workers in reducing the spread of COVID-19. Most of the internet interventions commenced after the COVID-19 outbreak as it exposed us to internet usage for meetings and training, which is still in use till date.

As stated by the NCDC, one critical priority for the response to the COVID-19 pandemic is scaling up of timely access to testing which was a huge challenge for Nigeria at the onset of the pandemic. Despite the efforts made to strengthen the country's surveillance system, it is still seen as poor and inadequate.^48^ Poor surveillance leads to underreporting of COVID-19 cases, their outcomes and deaths. The number of laboratory-confirmed cases is still relatively low compared to other countries in Africa and other continents. This might have resulted from low testing capacity experienced in the country. The lack of an active surveillance system and, where available, lack of sustained funds for logistics to carry out active surveillance contributed to this. In June 2020, Nigeria's capacity for COVID-19 testing was about 2,500 samples daily.^38^ However, only 50 per cent of this capacity was utilized due to constraints in the availability of testing kits, laboratories, and human resources. As of July 5th, 2021, only 0.012 per cent of the Nigerian population had been tested for the virus, 2,331,734 individuals in a population of over 200 million. This contrasts with South Africa, where more than 3.26 per cent of the population were tested for the virus, over 1.6 million individuals in a population of 58 million people.^38^

Other studies showed that efforts towards early detection and diagnosis would be better if time lags between tests and diagnosis were reduced to encourage more people to test and contain the spread of COVID-19.^50^,^51^ This was responded to through the deployment of electronic surveillance systems to laboratories to speed up the release of results and thus contributed to the number of cases detected so far, though not enough.

Vaccination is the best preventive method for spreading contagious disease; saw most countries struggle and plan to get the vaccines, curb the surge in cases, and protect people from the disease. Nigeria received its first COVID vaccines of 3.92 million doses of Oxford/AstraZeneca in March 2021. There was already a roll-out plan for this, with the frontline healthcare workers given the highest priority, followed by strategic leaders. The country also aimed to receive enough vaccines to cover 20% of its population by 2021. However, this was received with a high level of hesitancy by the populace, due to the misconceptions and rumors that followed the introduction of the COVID-19 vaccine, despite the reassurances by NAFDAC following a positive quality assessment.

This review has presented the need for more collaboration internationally and locally. Collaboration with local and international partners will be crucial to fast-track the acquisition of materials that can enhance quality service delivery, as the government cannot achieve optimal outcome alone. Sustaining the already existing collaborations/partnerships is essential for improved health systems.^51^

High political support was key to strengthening the capacity of the health system to contain the COVID-19 outbreak. Most interventions were initiated, implemented or supported by the government. However, more needs to be done to sustain these gains and further strengthen the health system to become resilient and withstand unexpected shocks. Measures such as strengthening the active surveillance system during health epidemics and sustaining partnerships will contribute towards strengthening health systems.

We, therefore, recommend that the health management information unit of FMOH and the NCDC should have a health systems response monitor that offers up-to-date information on the country's health system response to any epidemic/ pandemic and related public health initiatives, one that can be activated immediately any epidemic is confirmed and the health financing units should have budget for health security funds set aside for emergencies, as well as flexible national budget by the ministry of finance, budget and planning. We also recommend institutionalization of actions and processes to ensure clarity in roles, relationships and coordination mechanisms in health system governance by the health policy unit of the DHPRS department and across all levels of government regarding public health emergency responses. This can be done by reviewing and updating the national action plan and policy for health security (2018–2022) with the experiences of the COVID-19 response. There should also be adequate protection of infrastructure, equipment and materials acquired during this pandemic for future use by the custodians of these materials, with maintenance plans put in place for these.

### Limitations of the study

Although this assessment may not have identified all health responses in the published and gray literature despite attempts to be as comprehensive as possible, we opine that the findings can be viewed as accurate documentation of the situation in Nigeria. The sampling methodology and sample size may not permit broad generalization. Furthermore, the information generated may inform policies and strategies in Nigeria and other similar contexts. Further studies may complement our study findings.

## Conclusion

The COVID-19 pandemic placed a huge burden on the Nigerian health system and highlighted systemic weaknesses that prompted the Federal government of Nigeria to adopt a multi-sectoral response to the COVID-19 pandemic. These included implementations of interventions such as infection prevention and control measures, provision of equipment and resources for screening and diagnosis of viral infections, strengthening of health information management systems to produce timely data for health decision-making and service improvement, strengthening of the health system and increased health workforce capacity for improved service delivery for the populace. These measures motivated change in the behavior of health workers and consequently increased access and care for those diagnosed with COVID-19 and other health emergencies.

## Figures and Tables

**Figure 1:**
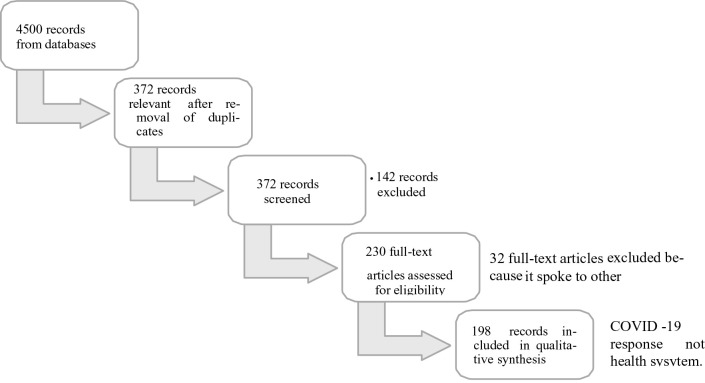
flow chart of documents used for rapid assessment

**Figure 2:**
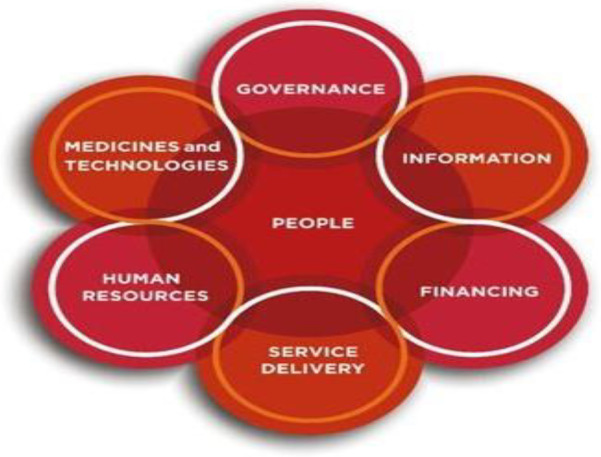
WHO Health systems building block (Adopted from WHO website).
